# Person-based design and evaluation of MIA, a digital medical interview assistant for radiology

**DOI:** 10.3389/frai.2024.1431156

**Published:** 2024-08-16

**Authors:** Kerstin Denecke, Daniel Reichenpfader, Dominic Willi, Karin Kennel, Harald Bonel, Knud Nairz, Nikola Cihoric, Damien Papaux, Hendrik von Tengg-Kobligk

**Affiliations:** ^1^Artificial Intelligence for Health, Institute for Patient-Centered Digital Health, School of Engineering and Computer Science, Bern University of Applied Sciences, Biel, Switzerland; ^2^Department of Radiology, Lindenhof Hospital, Bern, Switzerland; ^3^University Institute for Diagnostic, Interventional and Pediatric Radiology, Inselspital, University Hospital Bern, University of Bern, Bern, Switzerland; ^4^Department of Radiation Oncology, Inselspital, Bern University Hospital, University of Bern, Bern, Switzerland; ^5^Mimacom AG, Bern, Switzerland

**Keywords:** medical history taking, conversational agent, consumer health information, algorithms, patients, radiology, user-centered design, natural language processing

## Abstract

**Introduction:**

Radiologists frequently lack direct patient contact due to time constraints. Digital medical interview assistants aim to facilitate the collection of health information. In this paper, we propose leveraging conversational agents to realize a medical interview assistant to facilitate medical history taking, while at the same time offering patients the opportunity to ask questions on the examination.

**Methods:**

MIA, the digital medical interview assistant, was developed using a person-based design approach, involving patient opinions and expert knowledge during the design and development with a specific use case in collecting information before a mammography examination. MIA consists of two modules: the interview module and the question answering module (Q&A). To ensure interoperability with clinical information systems, we use HL7 FHIR to store and exchange the results collected by MIA during the patient interaction. The system was evaluated according to an existing evaluation framework that covers a broad range of aspects related to the technical quality of a conversational agent including usability, but also accessibility and security.

**Results:**

Thirty-six patients recruited from two Swiss hospitals (Lindenhof group and Inselspital, Bern) and two patient organizations conducted the usability test. MIA was favorably received by the participants, who particularly noted the clarity of communication. However, there is room for improvement in the perceived quality of the conversation, the information provided, and the protection of privacy. The Q&A module achieved a precision of 0.51, a recall of 0.87 and an F-Score of 0.64 based on 114 questions asked by the participants. Security and accessibility also require improvements.

**Conclusion:**

The applied person-based process described in this paper can provide best practices for future development of medical interview assistants. The application of a standardized evaluation framework helped in saving time and ensures comparability of results.

## 1 Introduction

Medical history forms the basis of clinical diagnosis and decision-making. A medical history interview should be conducted immediately before the investigation or on the same day. The medical history must be acquired frequently and, for some aspects, every time a person is exposed to examinations or interventions (Taslakian et al., 2016). Documentation from referring healthcare institutions is frequently not reliable and does not contain all necessary data items (Bell et al., 2020).

Computer-assisted history-taking systems or digital medical interview assistants (DMIA) are tools that help in obtaining relevant data on the medical history of patients (Pringle, 1988). Although such systems have been available for four decades, they remained unused in clinical routine (Slack et al., 1966). DMIA demonstrated to be efficient in saving professionals' time, improving delivery of care to those with special needs, and also in facilitating information collection, especially of potentially sensitive information (e.g., sexual history and alcohol consumption). Benefits of DMIA include the potential time saving since the patient history can be collected outside the patient-doctor encounter; the administrative burden of entering this information is reduced, patient face-to-face time is increased, and collected data can be automatically added to medical records available for automatic processing for decision support (Spinazze et al., 2021). Another positive aspect is that patients become more engaged in the diagnosis process resulting in improved participation in personal care, compliance with medication, adherence to recommended treatment, and monitoring of prescriptions and doses (Arora et al., 2014). Patient engagement becomes even more relevant with care concepts of value-based and patient-centered care. Good communication with patients has the potential to improve the coordination of care, improve safety and outcomes, increase patient satisfaction (Nairz et al., 2018) and decrease cost of care (Doyle et al., 2013).

Factors related to accessibility, affordability, accuracy, and acceptability have been identified as limitations of DMIA that hampered their adoption in daily routine (Spinazze et al., 2021). Acceptability challenges can originate in usability issues (Wei et al., 2011), i.e., users reported that they had difficulties in interacting with DMIA. Another limitation of existing tools is that irrelevant questions are posed by the system. Beyond, systems are difficult to use, resulting in frustrated users due to technical problems (Pappas et al., 2011). Research suggests that following a person-based approach in the design and development of a new system has potential to improve the system's quality and result in a higher level of user acceptance (Dabbs et al., 2009). Barriers toward the use of DMIA from a healthcare provider's perspective include (1) missing workflows and protocols related to patient-generated health data and (2) data storage, accessibility, and ease of use (Cohen et al., 2016).

In this paper, we are focusing on supporting the medical interview in the context of radiology by a DMIA that has been developed using a person-based approach and considering interoperability standards in healthcare to avoid the above-mentioned limitations of existing solutions. Radiology is a high-throughput medical discipline, highly dependent on and driven by complex imaging technology. These two factors, patient rush and technological advancement, have led to streamlined processes requiring very specialized labor skills. Hence, the radiology process is essentially bipartite, being split into an imaging and a reporting part. The interaction with patients to obtain images of internal body structures is generally performed by medical technicians, while the medical interpretation of images is under the responsibility of physicians. Thereby, the patient-physician relationship is disrupted (Rockall et al., 2022). Patients often do not know the role of radiologists, but they perceive value in consulting directly with imaging experts (Koney et al., 2016). Collecting the medical history in the context of mammography is crucial for several reasons. There are some physiological states or properties of a person that can significantly influence breast tissue and, therefore, impact the evaluation of an image by radiologists. For data on medical history, menopausal status, hormonal therapy or contraception, previous treatment, injuries, or symptoms may significantly impact imaging and change a radiologist's perspective (Jones et al., 2020; Han et al., 2022). For example, a vaccination can lead to swollen lymph nodes which can have an impact on the interpretation of the mammography image which makes information on a recent vaccination a relevant information from the patient's medical history. Information from the medical history can also lead to protocol changes for the radiological examination (Nairz et al., 2018). Neither the methodology of information transfer, nor the content of the medical history are currently considered optimal for supporting a radiologist in image interpretation (Nairz et al., 2018; Rockall et al., 2022). Based on the use case mammography/breast imaging, this paper describes a DMIA called “MIA,” implemented as a conversational agent that supports a radiologist in gathering accurate and current health information of a patient while giving the patient the opportunity to get answers to questions related to the examination. Conversational agents are software programs or components of larger systems designed to interact with users through natural language (Laranjo et al., 2018; Milne-Ives et al., 2020; Tudor Car et al., 2020). These agents feature complex technical properties, resulting in various types that span from rule-based systems with simple personalities to more sophisticated embodied agents with complex personalities (Denecke and May, 2023). Conversational agents can deliver information, answer questions, or assist with a range of tasks (Laranjo et al., 2018).

This paper describes the development process of MIA, its system architecture and the results from a comprehensive evaluation of the system including usability assessment.

## 2 Methods

We have already reported on the design process of MIA in a previous publication (see Denecke et al., 2023). Originally, MIA was only supposed to collect the medical history of a person before undergoing a radiological examination. We augmented this first system design with a dedicated module that enables MIA to provide answers to frequently asked questions regarding the examination. In this section, we briefly summarize the design and development of a testable prototype of MIA, and then focus on the evaluation methodology used to assess this prototype.

### 2.1 Design and development of MIA

#### 2.1.1 Requirements gathering

To ensure that the needs and perspectives of radiologists and patients are taken into account in MIA, the requirements engineering process was guided by a person-based approach as described by Yardley et al. (2015). This approach aims to embed iterative, in-depth qualitative research throughout the development process to ensure that the intervention is aligned with the psycho-social context of the end users. We also took into account the recommendations of the DISCOVER conceptual framework (Dhinagaran et al., 2022). DISCOVER provides a detailed protocol for the design, development, evaluation, and implementation of rule-based conversational agents. As a result, we established fundamental intervention goals to guide the development of MIA. These goals informed the specification of requirements, which were derived from a narrative literature review and a patient survey, and supplemented by specifications from a radiologist. The patient survey was distributed among members of the patient lobby group of a collaborating hospital (Inselspital Bern) comprising 25 members out of which 8 responded. The collected information was aggregated into functional and non-functional requirements for MIA. The requirement collection process was already described by Denecke et al. (2023), the list of requirements is made available (see data availability statement).

#### 2.1.2 Content generation

An initial set of 72 medical interview questions in German was defined by a single radiologist. In collaboration with two additional radiologists, this set of questions was reduced to 31 questions and augmented with allowable answers, forming a set of Common Data Elements (CDE). A CDE defines—the attributes and allowable values of a unit of information—and facilitates the exchange of structured information (Rubin and Kahn Jr, 2017). These CDEs were again iteratively improved before integration into MIA with respect to clarity, usefulness, relevance and correctness, as well as feasibility of technical implementation.

For the question answering (Q&A) module of MIA, we collected frequently asked questions related to mammography from information material provided by the Swiss national breast cancer screening program Donna (https://www.donna-programm.ch). Furthermore, we interacted with OpenAI ChatGPT to get additional inspiration for possible user questions, using the following prompt: “*Take the role of a woman undergoing a mammography for the first time. Which questions do you have regarding the examination.”* The resulting collection of question-answer pairs in German was reviewed, extended and corrected by two radiologists to ensure correctness and completeness. The questionnaire of the interviewer module and the Q&A module is made available (see data availability statement).

#### 2.1.3 System architecture

**Figure 1:**
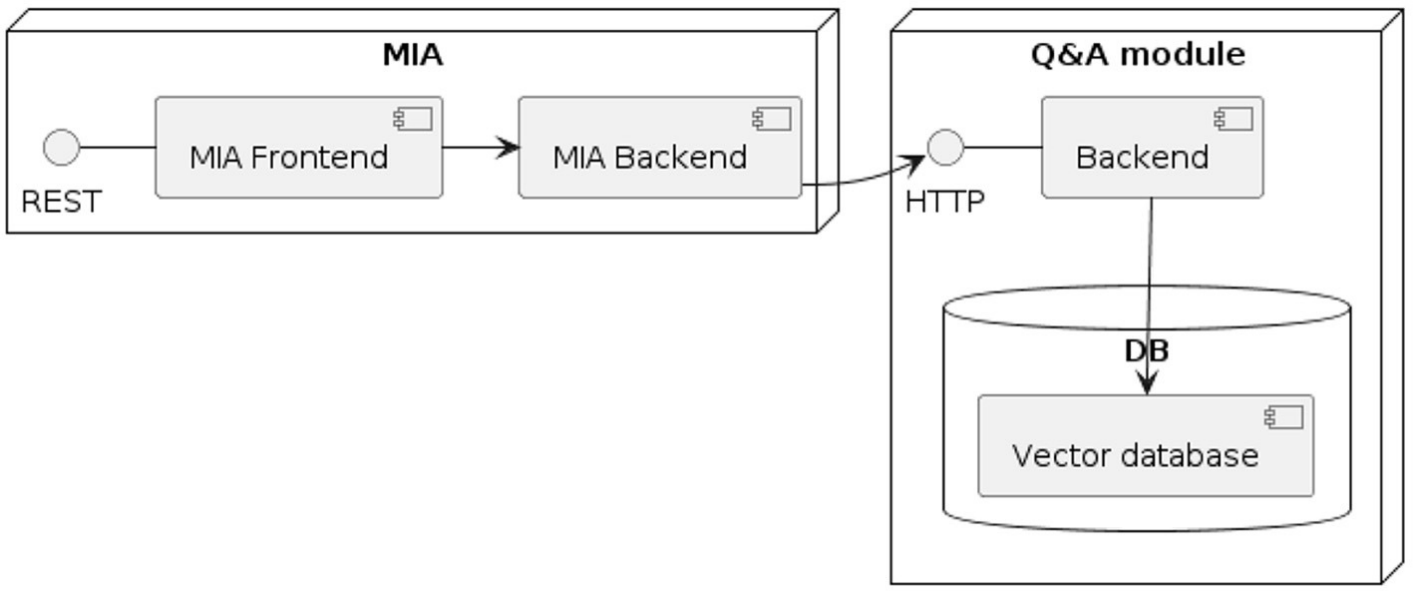
UML component diagram of a MIA instance (external RIS/HIS omitted).

The prototype of MIA includes two main modules (see Figure 1): First, there is the medical interview module, which is designed to work seamlessly with current hospital or radiology information systems. This central component features a web-based user interface that is specially optimized for use on tablets. Second, the Q&A module contains the logic that maps patient questions to pre-defined question-answer pairs.

The architecture of MIA was developed in a way that considers two major prerequisites: The architecture should allow to easily exchange the content of the conversational agent, i.e., the questions asked as part of the medial history interview. Beyond, the answers should be stored in a way that allows to import them into a hospital information system, thus ensuring interoperability. Both prerequisites are met by basing MIA on the Fast Healthcare Interoperability Resources (FHIR) standard for healthcare data exchange, published by Health Level Seven International (HL7). To implement FHIR, standardized data exchange formats, so-called FHIR profiles, were specified for defining the medical interview questionnaires and returning the resulting patient responses. A FHIR profile exactly specifies the type, cardinality, and structure of information to be persisted or exchanged between two systems. We based these profiles on the FHIR Structured Data Capture Implementation Guide, version 3.0.0 (SDC IG). The SCD IG is a FHIR-based framework that provides guidance related to filling in medical forms, comprising resource definitions and workflow considerations (HL7 International, 2023). Table 1 provides a description of the four profiles as well as their associated base profiles. The FHIR profiles are made available (see data availability statement).

**Table 1:** Description of the developed FHIR profiles.

**Profile**	**Derived from**	**Content**
MiaTask	SDCTaskQuestionnaire	MiaPatient resource, reference to URL location of questionnaire to be filled
MiaPatient	Patient	Full name and date of birth used for authentication
MiaQuestionnaire	SDCBaseQuestionnaire	Content and structure of medical interview
MiaQuestionnaireResponse	SDCQuestionnaireResponse	Answers to medical interview questions

As the Q&A module was added only late in the design phase, its data exchange formats were not specified. The Q&A module was not integrated into the MIA system, but is deployed independently and accessed via API call from the MIA system. Please refer to our previous publication, in which we describe the development process and evaluation of the Q&A module in detail (Reichenpfader et al., 2024).

#### 2.1.4 Information flow

The MIA system operates as a conversational agent, which means that the user interacts with the system through a dialogue. The dialogue flow of MIA consists of three distinct parts: On-boarding with authentication, the medical interview conducted by MIA and the question and answer part, where users can ask questions related to the examination. We describe the user interaction of the final system including the process triggering from the hospital information system in more detail below.

**Figure 2:**
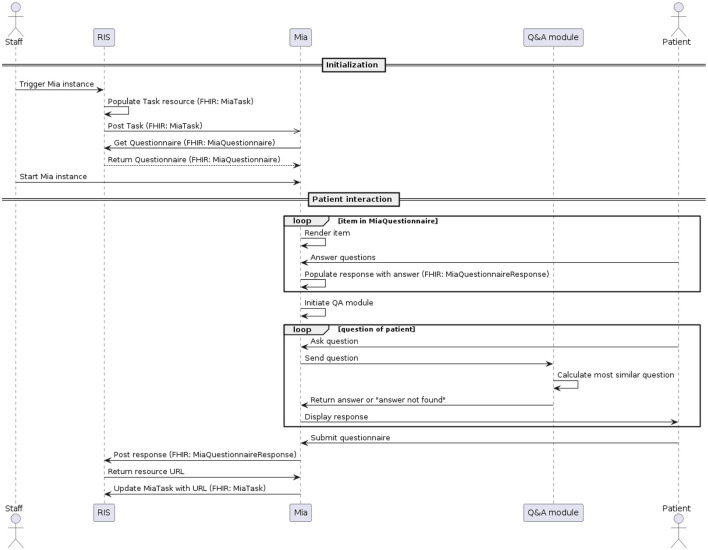
UML sequence diagram of an MIA interaction.

The MIA user interface is optimized for being accessed on a tablet, ideally hospital-owned. The process of conducting a medical interview for a certain patient, also called a task, is triggered within the hospital or radiology information system (see Figure 2). A MiaTask resource is sent to the MIA application, which then downloads the specified MiaQuestionnaire resource, containing the content and structure of the interview. Hospital staff see all open tasks on the tablet and trigger the start of a specific task before handing the patient the device. The device then displays the first part of the conversation flow, the on-boarding. For our usability testing, MIA did not communicate with external systems. One task and one questionnaire resource were hard-coded in the system and started by the test facilitator.

During the on-boarding process, users are welcomed by MIA, informed about how their data are handled, and asked to identify themselves by providing their full name as well as their birth date (in the usability test, pre-defined data was used). If they do not provide the right information as defined in the associated MiaPatient resource, the interaction process is suspended, and the patient is asked to reach out to hospital staff for help.

After successful identification, the MIA prototype renders a maximum of 31 questions about the patient's medical history, as defined in the MiaQuestionnaire resource. The questions concern previous visits to physicians and therapies related to the breast (chemotherapy, radiation therapy, etc.), and related to observations of recent changes in the breast region including pain or injuries. Eighteen questions allow for single-choice answers. Seven questions allow for multiple-choice answers and six questions are answered by entering free text. The system does not interpret free-text responses. Only responses to single- or multiple-choice questions change the conversation flow. For example, questions about pregnancy are only asked if the patient states not to be male. We make the MiaQuestionnaire resource used for usability testing available (see data availability statement).

In the third part of the dialogue flow, the user can ask free-text questions regarding the topic of mammography. Each patient question is individually sent to the Q&A module, which computes the most similar pre-defined question and returns the answer to MIA. The 33 predefined question-answer-pairs of the Q&A module, to which patient queries are made available (see data availability statement).

After finalizing the third part of the dialogue flow, a confirmation page is displayed with all questions asked by the user, as well as the responses they submitted. This allows the user to verify their responses before submitting their response. Currently, the answers shown in this summary cannot be edited. After the user submitted their responses, the system populates the MiaQuestionnaireResponse resource, containing the corresponding answers to the questions defined in the MiaQuestionnaire resource. This resource is then transmitted to the initiating system. For the usability test, the resource is not sent, but a log file is generated and downloaded locally instead. See Figures 1, 2 and for a UML component- and sequence-diagram of the system, respectively.

### 2.2 Evaluation of MIA

#### 2.2.1 Underlying evaluation framework

The evaluation of MIA was conducted based on the evaluation and development framework proposed by Denecke (2023a). The framework consists of four perspectives that in turn aggregate several evaluation categories:

- **Global perspective:** Accessibility, ease of use, engagement, classifier performance, flexibility, content accuracy, context awareness, error tolerance, and security.
- **Response understanding perspective:** Understanding.
- **Response generation perspective:** Appropriateness, comprehensibility, speed, empathy, and linguistic accuracy.
- **Aesthetics perspective:** Background, font, and buttons.

Furthermore, the framework suggests concrete metrics and heuristics to be used to evaluate a conversational agent in healthcare. We adapted the framework by removing the aspects that are not relevant for MIA. For example, from the aesthetics perspective, we removed the evaluation category “button” since MIA has no buttons. From the response generation perspective, we dropped “linguistic accuracy” since MIA does not generate answers, but simply posts phrases from the knowledge base. The complete set of evaluation aspects are listed in the Supplementary material (see data availability statement).

To evaluate MIA according to the heuristics and metrics described in the framework, we (1) conducted a technical evaluation of MIA (e.g., security aspects) using a design and implementation check, (2) assessed the usability using a task-based usability test and (3) analyzed conversation protocols as collected during the usability test.

#### 2.2.2 Study design and procedure for usability testing

The goal of the usability testing was to determine to what extent usability (efficiency, effectiveness, acceptance) is achieved by the current implementation as well as to identify aspects on how to improve the user interface and the conversation flow of MIA. The usability test was conducted under controlled test conditions with representative users. Prior to participant recruitment, the study plan underwent review by the regional ethics committee and was determined to be exempt from approval (BASEC-Nr: Req-2023-00982).

We aimed at recruiting a total of 30 patients undergoing a mammography for the usability testing from two collaborating hospitals. According to usability expert Jacob Nielsen, “testing with 20 users typically offers a reasonably tight confidence interval” within usability testings (Nielsen, 2006).

The usability test was conducted in a closed room within each respective hospital. In addition to these patients, we recruited members of two different patient organizations to join the usability test. Their usability test was following the same procedure as the patients, except that they were not undergoing a mammography examination afterward and they were answering an additional questionnaire with heuristics. The following exclusion criteria for participant recruitment were defined:

- No basic skills with interacting with a smartphone or tablet.
- Unwillingness to interact with MIA.
- No knowledge of German language of at least B1 level.
- Patients who are unable to read or write.
- Patients younger than 18 years.

The participants did not receive any monetary compensation, but a small box of chocolate. The usability test comprised two tasks: First, participants were instructed to answer the questions that are asked by MIA, followed by asking at least three questions to MIA related to an upcoming mammography examination. The participants were asked to think aloud and to provide honest opinions regarding the usability of the application, and to participate in post-session subjective questionnaires and debriefing. Below, we describe the test procedure in detail. Each test session was conducted in a separate room to ensure privacy and accompanied by a facilitator, who:

- provided an overview of the study and the system to participants,
- defined the term *usability* and explained the purpose of usability testing to participants,
- assisted in conducting participant debriefing sessions,
- responded to participant‘s requests for assistance, and
- collected the comments provided by the participants during the testing and during the post-testing interview.

Upon agreement to participate in the test, each participant was assigned a random identifier and provided with a test device (Apple iPad Pro 2018). First, participants filled in the first part of the online questionnaire, which was created using a local LimeSurvey instance. This first part comprised demographic information as well as questions to validate fulfillment of any exclusion criteria, namely knowledge on the topic of mammography, whether the person has already had a mammography before, gender, age, familiarity with tablet and mobile phone use and self-judgment of German language skills.

If the participant was not excluded, they continued with the actual usability test. To ensure anonymous data collection, each participant was provided with the same fictitious name and date of birth. In this way, all data was collected anonymously. After the completion of both tasks, the participant was provided with the second part of the online questionnaire, comprising standardized questionnaires to be rated on a 5-item Likert Scale. We applied the Bot Usability Scale as described by Borsci et al. (2022). Additionally, we added eleven additional questions that were part of the evaluation framework (Denecke, 2023a). These questions refer to empathy expressed by MIA, comprehensibility and perception of the capabilities of MIA as well as aesthetic aspects such as background color and font type.

The members of patient organizations additionally assessed MIA based on eleven heuristic criteria for conversational agents in healthcare proposed by Langevin et al. (2021). For each heuristic, we defined a concrete catalog of criteria for assigning 1, 2, or 3 points per item. The heuristics and the criteria can be found in the Supplementary material.

#### 2.2.3 Data analysis

To ensure an unbiased data analysis, the data collected in the user study was analyzed by two authors (DW, KK) who were neither involved in the development of the system nor involved in the treatment of the participants. We collected the data from the conversation protocols (i.e., the interaction between MIA and participant), the usability questionnaires and the notes taken by the facilitators.

## 3 Results

### 3.1 Results from the technical evaluation

In the following, we summarize the results of the technical evaluation that resulted from a design and implementation check. In Section 3.2, we report all results that have been collected within the usability testing. The complete list of results of the evaluation framework is available in the Supplementary material.

#### 3.1.1 Accessibility

The readability of MIA's content was calculated using four different readability scores: SMOG (Simple Measure of Gobbledygook) Readability, Gunning Fog Index, Flesch Reading Ease Score and LIX (Fabian et al., 2017). While Flesch Reading Ease Score, Gunning Fog Index and SMOG consider syllables and unfamiliar words for their calculation, LIX calculates the percentage of words with seven or more letters, i.e., it calculates the index by considering the number of sentences and the number of long terms. Table 2 summarizes the scores for the interview module and Q&A module.

**Table 2:** Results from readability assessment.

**Readability index**	**LIX**	**Flesch Reading Ease Score**	**Gunning Fog Index**	**SMOG**
Tool used	https://www.supertext.ch/tools/lix	https://www.flesch-lesbarkeitsindex.de	https://charactercalculator.com/gunning-fog-index/	https://charactercalculator.com/smog-readability/
Interview module	67	64	11.15	64
Q&A module	51	46	17.46	46

The content of the Q&A module reaches an average LIX value of 51/100 which corresponds to a language level of C1 (Common European Framework of Reference for Languages). The interview module has a readability index of LIX 67/100, corresponding to language level C2. The other scores provide a slightly different picture. In these assessments, the content of the Q&A module is recognized as rather complex to be understood. A Flesch Reading Ease Score of 46, a Gunning Fog Index of 17.46 and a SMOG of 46 for the content of the Q&A module correspond to college or undergraduate reading level, i.e., difficult to read. For the interview module, a LIX of 64 and SMOG of 10.45 correspond to plain English, to be easily understood by 13–15 year old students. A Gunning Fog Index of 11.15 corresponds to 11th grade, i.e., fairly difficult to read.

MIA does not provide alternatives for written input or output. The contrast between text and background color is 3.6:1. It is possible to resize text in the graphical user interface. Accessibility guidelines have not been considered in the development phase.

#### 3.1.2 Content accuracy

The underlying knowledge base of MIA is evidence-based and healthcare professionals as well as representatives of patient organizations were involved in the development process. However, a maintenance process for MIA's content has not yet been developed as the current prototype is considered a proof-of-concept implementation. Information on the developer and content provider is shown to the user during the on-boarding process.

#### 3.1.3 Context awareness

Context awareness is not given in the current implementation of MIA; context switches are not recognized because of the realization of MIA. User input in the interview module is only used to decide whether a follow-up question is asked or not.

#### 3.1.4 Flexibility in dialogue handling

Flexibility in dialogue handling is not yet provided by MIA given its rule-based implementation and the missing interpretation of user input. The interview module asks only one question after another as foreseen in the pre-defined conversation flow. In the Q&A module, the user query is matched with the knowledge base. When no match can be found, a standard answer is provided.

#### 3.1.5 Security

Only few security measures are implemented in the prototype: User authentication, authorization, and session management were implemented. A privacy statement is provided to the user in the on-boarding process. Standard operating procedures are in place for processing personal identifiable information according to the privacy statement. MIA is compliant with the current regulations about data privacy which is the general data protection regulation in Europe. The programming packages used in MIA are scanned for vulnerabilities. For the development of MIA, no security-by-design approach was followed and no established security management standard was applied. No measures have been implemented for managing reliability and maintenance of third party software and components used. Additionally, collected data is not encrypted and no process has been established yet to test the security of MIA on a regular basis.

#### 3.1.6 Technical issues

Technical issues were collected using the notes made by the facilitators during the tests. They were grouped into high priority, low priority, additional requirements and additional aspects mentioned. Five issues were classified high priority. For example, the entry of the date of birth was suggested to be facilitated. Three items were of low priority, e.g., to improve the drop down menu. Specifically, a drop down list is redundant when only one out of two items can be selected. Two additional requirements were collected: It was suggested to add a button for requesting talking to a human and to display the patient name in the chat view. FOur additional aspects were mentioned, e.g., that it was unclear, in which order name and surname have to be entered for authentication.

### 3.2 Results from the usability test

#### 3.2.1 Participants

We included 36 participants in the usability test. 30/36 participants were actual patients interacting with MIA before undergoing their mammography examination. 6/36 were members of patient organizations. They filled an extended form of the usability questionnaire including heuristic criteria. The tests were conducted on 5 days between December 2023 and February 2024.

Four participants were between 40 and 49 years old (11.1%); 12 (33.3%) between 50 and 50 years; nine participants (25%) were between 60 and 69 years old and 11 participants 70 years or older (30.5%). The majority of participants were women (32 participants, 88.9%). Thirty-five participants (97.2%) were native speakers in German, one participant selected language level B2. Two participants had no previous experience with smartphones and tablets (5.5%). Out of the individuals surveyed, 30 (83.3%) had undergone a mammography at some point earlier in their lives. Four individuals had little to no knowledge about mammography. Three participants had only a minimal understanding of the topic. Nineteen individuals possessed basic knowledge about mammography, while 10 participants were very familiar with the subject.

#### 3.2.2 Results from the heuristic evaluation

**Figure 3:**
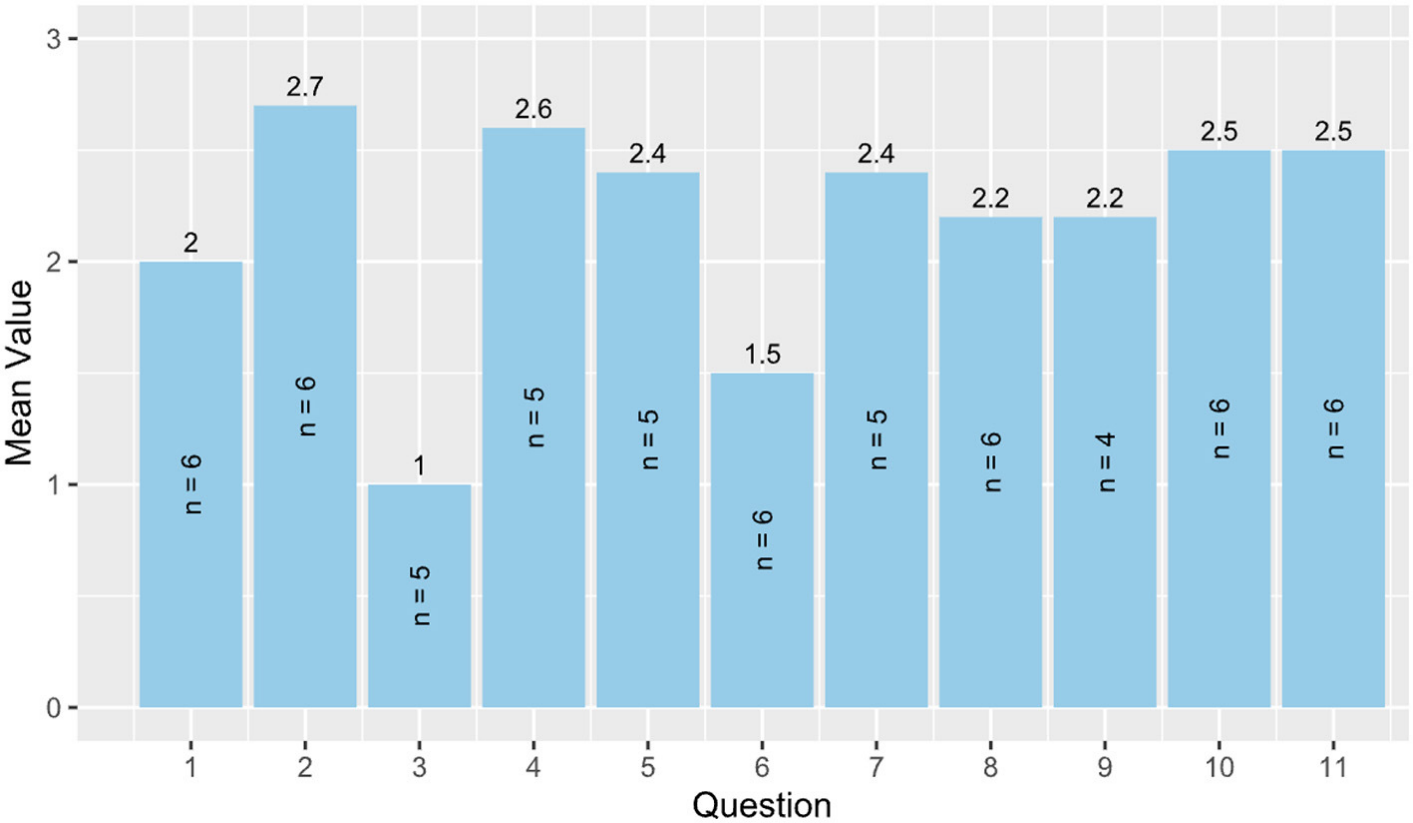
Heuristic evaluation. 1 = Visibility of system status, 2 = Match between system and real world, 3 = User control and freedom, 4 = Consistency and standards, 5 = Error prevention, 6 = Help and guidance, 7 = Flexibility and efficiency of use, 8 = Aesthetic, minimalist, and engaging design, 9 = Help users recognize, diagnose, and recover from errors, 10 = Context preservation, and 11 = Trustworthiness.

Results from the heuristic evaluation are shown in Figure 3, *n* = 6. Since the questions were not mandatory, four questions were only answered by 5/6 participants and one question by 4/6 participants. All other questions were answered by six participants. It can be seen that there is still potential for improving user control and freedom (question 3) where the smallest mean values were achieved. Furthermore, help and guidance (question 6) shows potential for improvement. The other items achieved mean values of 2 and above. The sum of the mean values is 24 out of a maximum of 33 points.

#### 3.2.3 Usability questionnaire

**Figure 4:**
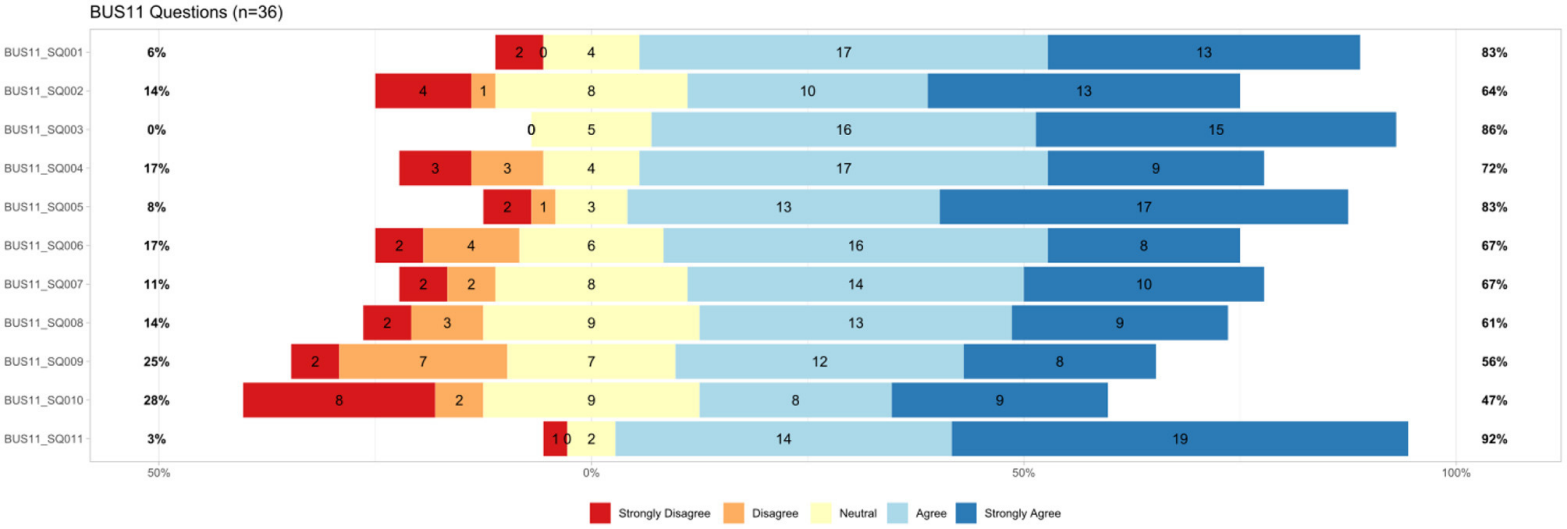
Results from BUS-11 questionnaire (Bot usability scale), *n* = 36, BUS11_SQ001=“The chatbot function was easily detectable.” BUS11_SQ002=“It was easy to find the chatbot.” BUS11_SQ003=“Communicating with the chatbot was clear.” BUS11_SQ004=“The chatbot was able to keep track of context.” BUS11_SQ005=“The chatbot's responses were easy to understand.” BUS11_SQ006=“I find that the chatbot understands what I want and helps me achieve my goal.” BUS11_SQ007=“The chatbot gives me the appropriate amount of information.”, BUS11_SQ008=“The chatbot only gives me the information I need.” BUS11_SQ009=“I feel like the chatbot's responses were accurate.” BUS11_SQ010=“I believe the chatbot informs me of any possibly privacy issues.” BUS11_SQ011=“My waiting time for a response from the chatbot was short.”

The questionnaire for the Bot usability scale (BUS-11) was answered by 36 participants. Results are shown in Figure 4. The BUS-11 questionnaire is provided as Supplementary material (see data availability statement). Perceived accessibility to the chatbot function was good (BUS11_SQ001 and SQ002). The system does not provide any other functions than the chatbot. Perceived quality of chatbot functions consists of three questions (BUS11_SQ003-5) that were to be judged by the participants. Eighty-six percentage agreed with the statement that communication with the chatbot was clear. Seventy-two percentage agreed that MIA was able to keep track of the context. 83% confirmed that MIA's responses were easy to understand. Perceived quality of conversation and information provided (BUS11_SQ006-9) shows potentials for improvement. Sixty-seven percentage agreed and find that the chatbot understands what they want and helps achieving the goal. Sixty-seven percentage think the chatbot provides them with the appropriate amount of information. Sixty-one percentage participants agreed with the statement that the chatbot only gives the information needed. Fifty-six percentage had the impression the chatbot answers were accurate. Perception of privacy and security was limited (BUS11_SQ010): Only 47% agreed that they believe MIA informs them of any possible privacy issues. Time response (BUS11_SQ011) was short as stated by 92% of the participants.

**Figure 5:**
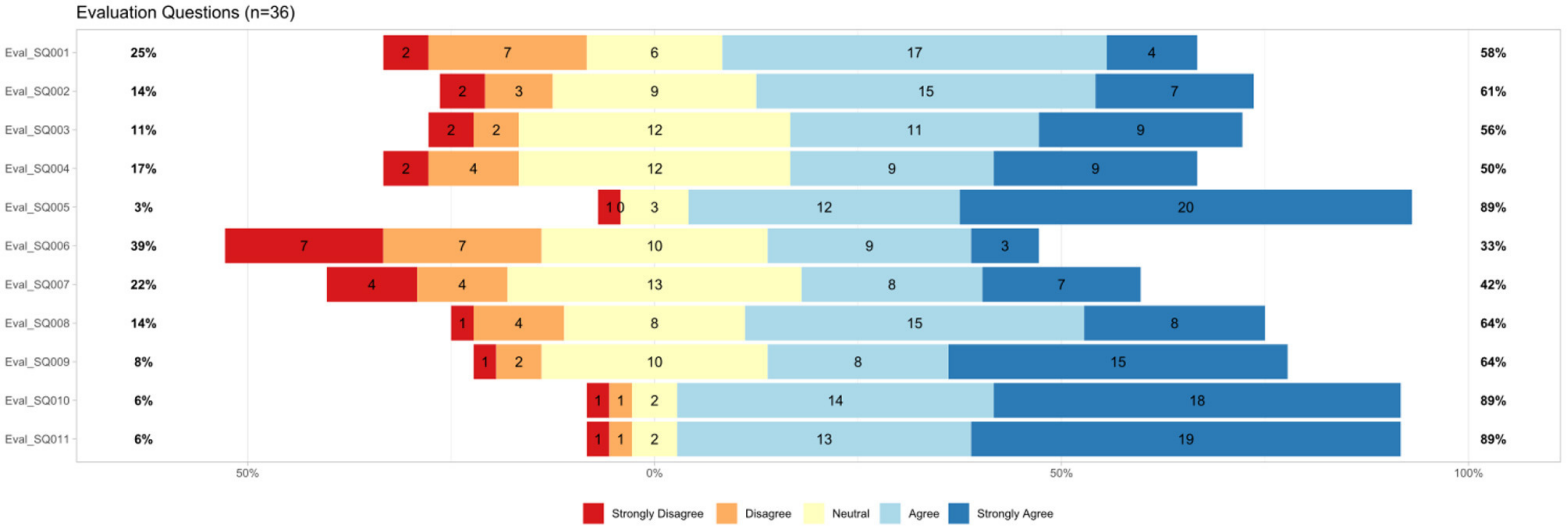
Results from additional usability questions (n = 36), Eval_SQ001=“I have the impression that the digital assistant understands what I want to know.” Eval_SQ002=“I have the impression that the digital assistant helps me to get answers to my questions about the examination.” Eval_SQ003=“The digital assistant provides me with an appropriate amount of information.” Eval_SQ004=“I have the feeling that the digital assistant's answers are tailored to my needs.” Eval_SQ005=“The waiting time for a response from the digital assistant was in line with my expectations.” Eval_SQ006=“The digital assistant did not recognize how much I was bothered by some of the things discussed.” Eval_SQ007=“The digital assistant understood my words but not my feelings.” Eval_SQ008=11 “I think the digital assistant generally understood everything I said.” Eval_SQ009=I like the background color.” Eval_SQ010=“The font was easy to read.” Eval_SQ011=“The font size was appropriate for me.”

The results of the additional questions on usability aspects are shown in Figure 5. Font type and size were perceived well. Eighty-nine percentage participants agreed that the font was easy to read and the size was appropriate (Eval_SQ010 and SQ011). Six percentage disagreed with these statements. Sixty-four percentage liked the background color while 8% disliked it (Eval_SQ009).

There were additional questions asked related to comprehensibility and understanding. Sixty-four percentage think that MIA generally understood them; 14% did not confirm this (Eval_SQ008). Forty-two percentage had the impression that MIA was understanding them, but not their feelings (Eval_SQ007). Twenty-two percentage disagreed with that statement. Further, 33% confirmed that MIA did not recognize how much they were bothered by some things discussed (Eval_SQ006). Thirty-nine percentage disagreed with that statement. The waiting time for response was within expectation for 89% of the participants in contrast to 3% who disagreed (Eval_SQ005).

The answers were perceived as tailored to user needs by 50% of the participants; 17% disagreed (Eval_SQ004). Fifty-six percentage participants agreed that the appropriate amount of information was provided by MIA; 11% disagreed (Eval_SQ003). Additionally, 61% agreed that MIA helps to get answers on the examination, while 14% disagreed with that statement (Eval_SQ002). Fifty-eight percentage of the participants had the impression, MIA understands what they want to know; 25% neglected this (Eval_SQ001). The results are shown in Figure 5.

In the verbal feedback, one participant claimed that the amount of text was too large. Texts should be rather split into several chunks. All questions asked by MIA were mandatory, which was not perceived well. MIA was perceived as not empathetic since the system did not address comments such as “I do not feel well.” It was suggested that MIA could guide better through the different topics for example by providing some information on the topic of the coming questions (e.g., “Now, I will ask questions on wellbeing”). During the interview, MIA asks whether the situation of the patient improved since the last visit to a doctor. Regarding this question, one participant stated that this is difficult to judge when the visit was more than 2 years ago. Some questions were perceived as useless in the context of mammography.

#### 3.2.4 Analysis of interaction with Q&A module

While 36 usability questionnaires were analyzed, only 35 interaction protocols could be analyzed since one interaction was excluded. This participant was seriously visually impaired and the interaction with MIA was done by one of the facilitators who read the questions asked by MIA and entered the answers from the participant.

**Figure 6:**
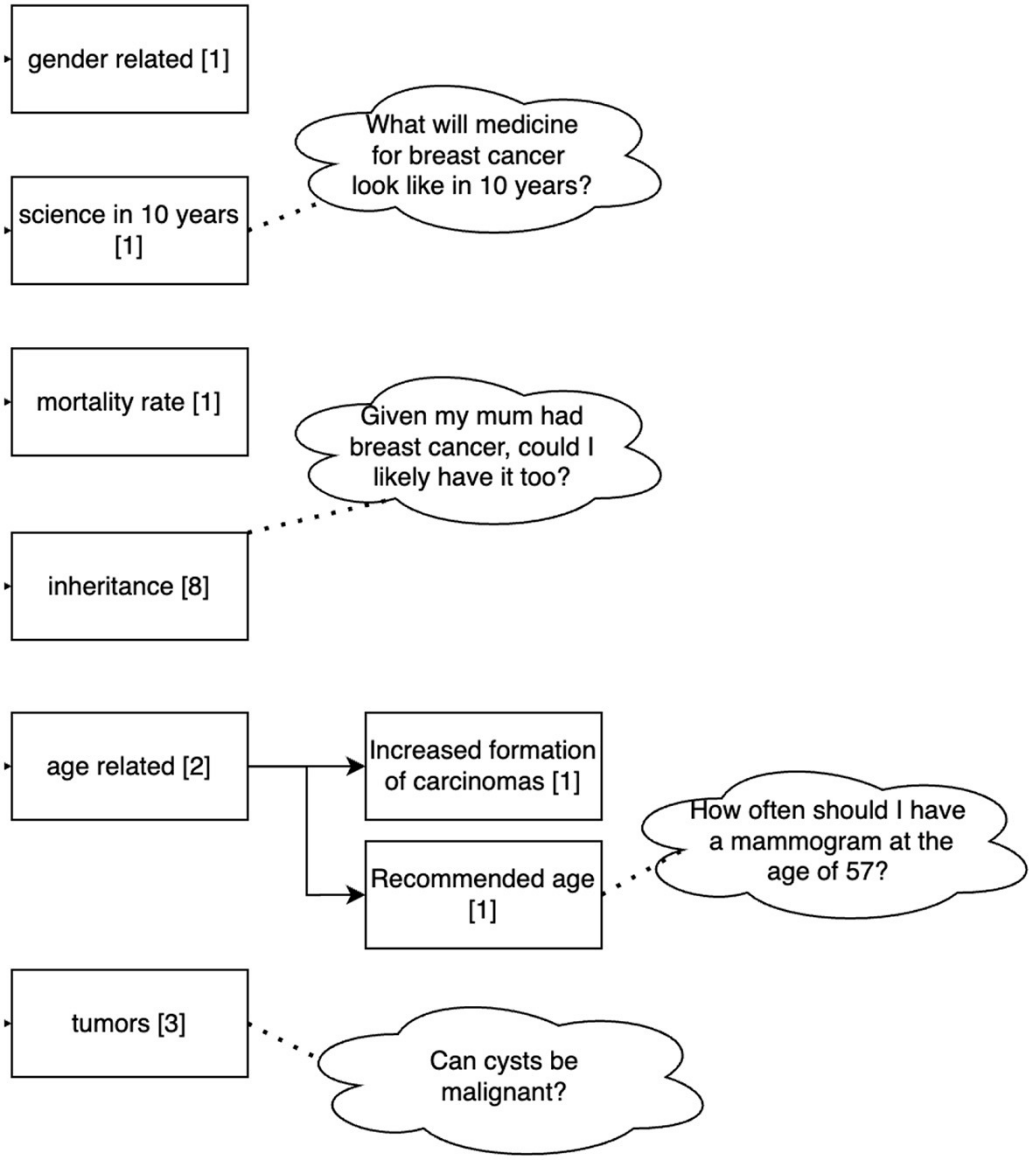
Clustering of the queries related to the disease asked by the 36 participants to the Q&A Module. Sixteen out of 114 questions dealt with the disease. Numbers in brackets refer to the number of questions belonging to this cluster.

**Figure 7:**
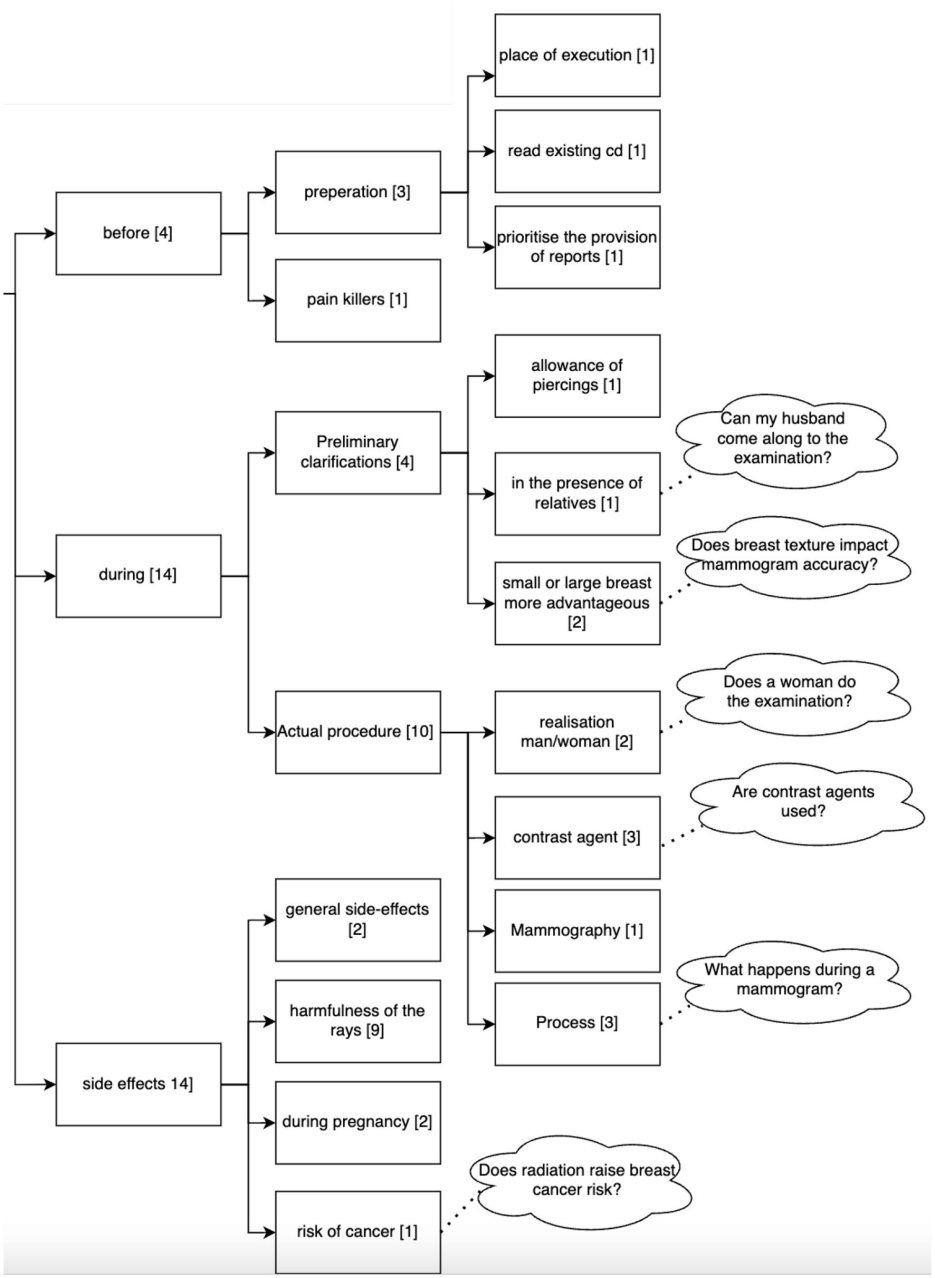
Clustering of the queries asked by the 36 participants to the Q&A Module related to the procedure of a mammography. Numbers in brackets refer to the number of questions belonging to this cluster (*n* = 114).

A total of 114 questions related to the topic of mammography were asked by the 36 participants to the Q&A module. The questions can be divided into two main topics: disease-related (16 questions) and examination-related (Mammography, 92 questions). Questions related to the disease (breast cancer) can be grouped into gender-related, scientific perspectives related to the treatment (e.g., how will medicine for breast cancer look like in 10 years), mortality rate, inheritance of the disease, questions on tumors and age-related questions (see Figure 6). Questions related to the examination addressed its duration, frequency, age, costs, sensation of pain, results and aspects related to the procedure. Figure 7 shows the clustering of the queries related to the procedure of the examination. It can be seen that most questions referred to aspects during the examination and possible side effects.

**Figure 8:**
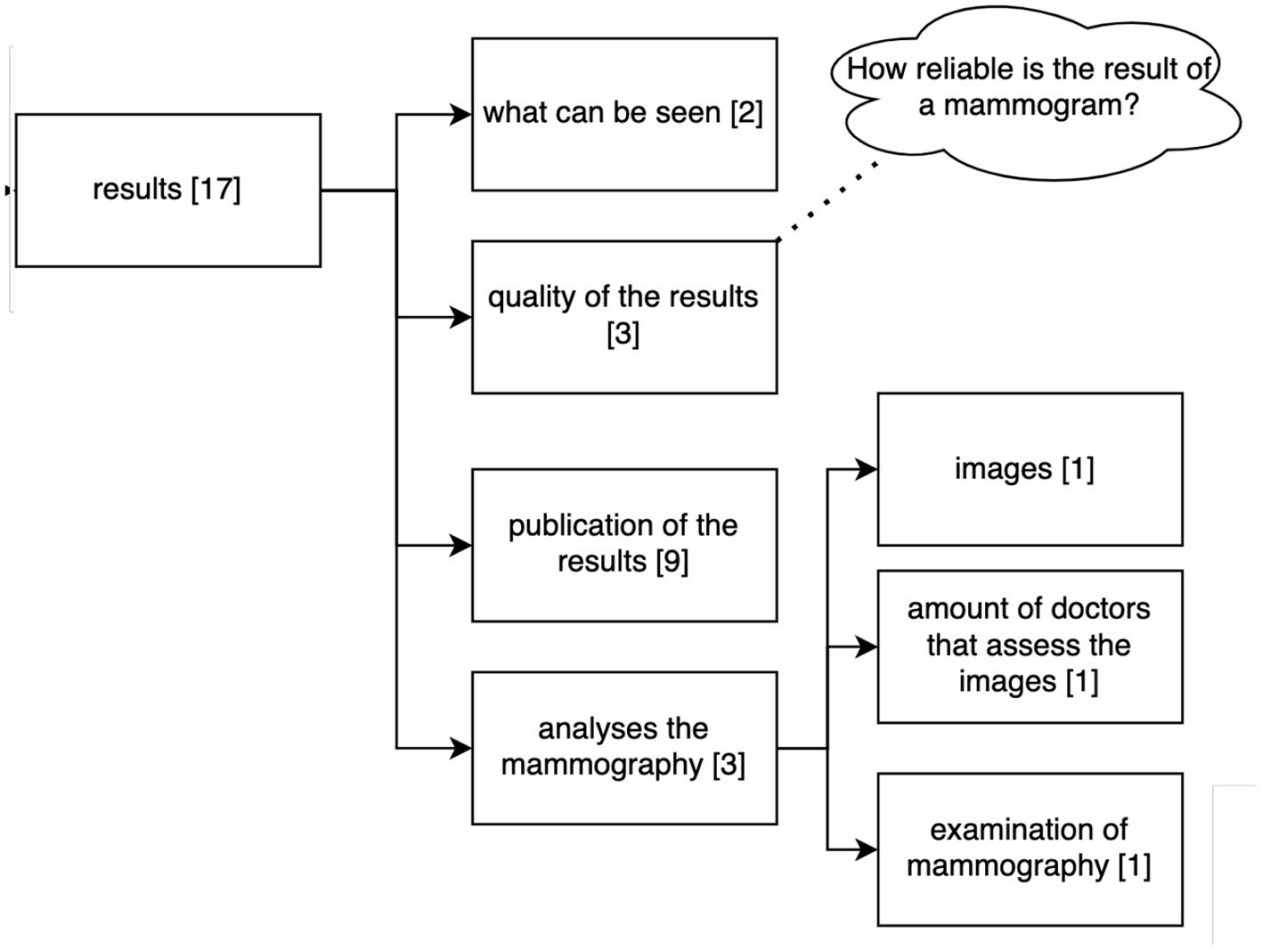
Clustering of the queries asked by the 36 participants to the Q&A Module referring to the results of the mammography. Numbers in brackets refer to the number of questions belonging to this cluster (*n* = 114).

Figure 8 shows the clustering of the queries related to the results collected by the examination. Participants asked about aspects that can be seen in the mammogram, the quality of the results for diagnosis, publication of the results and analysis related to the mammogram.

The evaluation showed that MIA's Q&A module is not flexible in dialog handling, basically because of its matching algorithm based on question similarity. In case a patient question is not similar enough to a pre-defined one, a fallback mechanism is triggered and no answer is given. The question asked must contain more than 3 words for the module to provide an answer. MIA can not handle shorter queries.

The Q&A module achieved a precision of 0.51, a recall of 0.87, an *F*-score of 0.64 and an accuracy of 0.54. This corresponds to 47/114 true positive answers (question part of the knowledge base and correctly answered, 46/114 false positives (question not part of the knowledge base, but answered by MIA), 7/114 false negative answers (question part of knowledge base, but not answered), and 14 true negatives (question not part of the knowledge base and not answered).

## 4 Discussion

### 4.1 Principal findings

We designed, developed, and evaluated a prototype of a medical interview assistant for radiology for the concrete use case of collecting information from patients before undergoing a mammography. Unlike other DMIAs, our system can render any definition of medical interview questions as long as they follow the defined FHIR profile. Furthermore, MIA allows patients to ask questions on the examination they are supposed to undergo. We conducted a comprehensive usability test with 36 participants. Comparable studies only include 10 participants, as for example the study on a hypertension self-management chatbot described by Griffin et al. (2023). A specific strength of MIA is its standardized format for exchanging medical interview questions and associated patient answers, based on HL7 FHIR. To the best of our knowledge, this is the first implementation of a medical interview assistant as conversational agent that allows to transfer the collected information in HL7 FHIR format and thus ensures seamless interoperability with clinical information systems.

The Q&A module provides a benefit for patients who can ask their questions related to the examination. It remains to the future to assess whether this component improves also patient's satisfaction with the examination process, whether possible fears can be reduced and whether they feel better informed. Another strength of the developed system is that the knowledge base of the Q&A module is designed to be improved over time: Patient questions that cannot be answered by the system are stored. These unanswered questions can periodically be reviewed by physicians, who then add a corresponding answer to the system. Thus, the knowledge base is extended and adapted to real-world patient needs on an ongoing basis.

The rather low precision of 0.51 achieved in the usability test for the Q&A module might be due to the following two reasons: First, the initial knowledge base created for usability testing consists of only 33 question-answer-pairs. Adding additional categories of questions as well as alternative formulations of existing question-answer-pairs might increase the true positive rate and decrease the true negative rate. Second, the required similarity threshold for an answer to be matched is set to 0.7 (cosine similarity). By gradually increasing this threshold, the false positive rate might be reduced until false negative rate increases.

Although recent developments including large language models are showing great potential in various healthcare settings (Denecke et al., 2024), MIA was purposely designed as a rule-based agent to limit the shortcomings and pitfalls that conversational agents can lead to Denecke (2023b). While this design decision caused less flexibility in the conversation flow, the major advantage is the gained control over the system and the avoidance of misinformation: Neither during the medical interview nor when answering questions the system is going to hallucinate, making up answers or providing clinically wrong responses. Therefore, there is no risk of misinformation since the complete knowledge base was provided by clinical experts. However, we see a future application of large language models in the context of MIA, namely in improving accessibility: A large language model could be used to tailor the standardized and pre-defined content of MIA to the specific needs of the patient. This could include providing explanations or re-formulate statements in the language preferred or better understandable by an individual user.

Based on a test with real patients in the setting as the system is supposed to be used, we achieve a technology readiness level (TRL) of 6 for our implementation (with TRL 9 as maximum, defined as actual system proven in operational environment). However, several aspects still have to be considered before applying MIA for information collection in a treatment setting. Using HL7 FHIR allows to integrate the collected information into a clinical information system - however, we have not yet tested this. Moreover, HL7 FHIR has not been widely adopted yet. Accessibility still has to be improved: The system uses large amounts of texts that might not be well-understood by all patients. The readability assessment shows that the language is quite complex. One participant claimed that there is too much text to read. A voice input or output has not yet been realized, which means that visually impaired persons are excluded from the usage. Such a situation occurred during the usability test and demonstrated the need for ensuring accessibility. We plan to address these issues in future by considering principles of inclusive design.

The evaluation showed that the aspects of perceived privacy and security have to be improved. This is essential for achieving user acceptance of such an interview assistant given the fact that privacy of patient-doctor communication is protected by law. Also important for trustworthiness is the perceived quality of the system by its users - in this regard, MIA still has potential for improvements. In particular the Q&A module has to be improved to be able to answer more patient questions in an acceptable manner. Steerling et al. (2023) found in their study that individual characteristics, characteristics of the artificial intelligence-based solution and contextual characteristics influence on user's trust. Nadarzynski et al. (2019) confirmed that transparency of the chatbot system regarding its quality and security is important for engagement and trust of users. Interestingly, some questions asked by the medical interview module were perceived as redundant by the users in the context of mammography (e.g., a question on vaccinations). In the context of an actual patient-doctor encounter, a physician could explain why the information is relevant.

### 4.2 Reflections on the application of the evaluation framework

In this work, we used an existing framework proposed to evaluate conversational agents in healthcare (Denecke, 2023a). We conclude that the framework is useful to evaluate a chatbot from a technical perspective. The provided metrics and methods can be easily applied and we saved time in designing the evaluation procedure by relying upon the specified metrics and methods. It also helped in reducing time for developing the evaluation questionnaire. We had to recognize that some questions are quite similar, resulting in participants asking for clarification.

The framework is very comprehensive and when applying it to our system, we had to select the aspects that are of relevance. We had to drop 19 metrics since they were redundant (e.g., button color). However, we believe that it is better in terms of comparability to drop metrics instead of adding new ones and, in this way, loose comparability of evaluation results. In our case, application of the framework was slightly challenging, since the system consists of two modules that are realized differently that could have been even considered as individual conversational agents because of their different technical implementation and purpose. The interview module only asks questions without any interpretation. The Q&A module does initiate the conversation, but only replies to a question asked by a patient. However, we decided to consider both modules as one system as they are perceived by the users as one system. For the usability test, we applied a set of standard tools (BUS-11, Borsci et al., 2022 and heuristics, Langevin et al., 2021). The test sessions showed that some items of the BUS-11-scale were not relevant for our system, but to be comparable with other assessments that use BUS-11, we did not remove them. For example, one item is “*It was easy to find the chatbot”*—our system only consists of the chatbot; there is no way to miss it since it starts as soon as the web page has been opened.

Applying the framework at an earlier stage of the development process could have helped in addressing the accessibility and security aspects right from the beginning. However, the evaluation using the framework clearly showed what has to be done to be able to use the system in real world. The evaluation results will thus help us to improve the system in the next iteration of development.

### 4.3 Limitations

Our evaluation has some limitations. The sample was limited to participants from two hospitals and some participants from two patient organizations. All participants had good German language skills: most of them were German native speakers. This might limit the generalizability of the results in terms of understandability. Furthermore, the analysis of the readability using the LIX Readability index has limitations: It considers the number of sentences when calculating the index. We entered the complete set of queries asked by MIA to the calculation platform. It would have been better to determine the index for each statement and calculate the mean value of the indices. To address this issue, we applied three other readability scores. Altogether, they provide a clear picture of the readability of the text provided by MIA.

## 5 Conclusion

In this paper, we introduced a medical interview assistant with a conversational user interface for radiology. We provided an overview of our design and development process, which may provide best practices for the person-based development of such systems. In particular, we make the FHIR profiles available and we recommend their application in similar systems to foster interoperability. We applied a comprehensive evaluation framework to study the quality of all relevant technical aspects of the system. The results can serve as a benchmark for future implementations of medical interview assistants with conversational user interface. Given the increased interest in collecting patient-reported outcome measures as quality indicators, our work may pave the way to collect such measures using a conversational agent. This might provide an improved user experience for some patient groups. In future work, the relevance of the collected information for the diagnostic process will be studied. We will improve the system following the potentials for improvement derived from the evaluation and user testing. A content maintenance process will be developed to allow for quick adaptations of the questionnaires to other examinations or even for the collection of patient-reported outcome measures.

## Data availability statement

The original contributions presented in the study are available https://doi.org/10.17605/OSF.IO/6MBHD (MIA questionnaire and FHIR resources), https://doi.org/10.5281/zenodo.10782322 (code for the chatbot) and are included in the article/Supplementary material, further inquiries can be directed to the corresponding author.

## Ethics statement

Prior to participant recruitment, the study plan underwent review by the Regional Ethics Committee (Kantonale Ethikkommission, Kanton Bern) and was determined to be exempt from approval (BASEC-Nr: Req-2023-00982). The studies were conducted in accordance with the local legislation and institutional requirements. Written informed consent for participation was not required from the participants or the participants' legal guardians/next of kin in accordance with the national legislation and institutional requirements.

## Author contributions

KD: Conceptualization, Methodology, Visualization, Writing – original draft, Writing – review & editing. DR: Investigation, Software, Visualization, Writing – original draft, Writing – review & editing. DW: Investigation, Writing – review & editing. KK: Investigation, Writing – review & editing. HB: Resources, Writing – review & editing. KN: Project administration, Writing – review & editing. NC: Resources, Writing – review & editing. DP: Software, Writing – review & editing. HvT-K: Resources, Writing – review & editing.
