# Corrigendum: Person-based design and evaluation of MIA, a digital medical interview assistant for radiology

**DOI:** 10.3389/frai.2024.1546421

**Published:** 2025-01-07

**Authors:** Kerstin Denecke, Daniel Reichenpfader, Dominic Willi, Karin Kennel, Harald Bonel, Knud Nairz, Nikola Cihoric, Damien Papaux, Hendrik von Tengg-Kobligk

**Affiliations:** ^1^Artificial Intelligence for Health, Institute for Patient-Centered Digital Health, School of Engineering and Computer Science, Bern University of Applied Sciences, Biel, Switzerland; ^2^Department of Radiology, Lindenhof Hospital, Bern, Switzerland; ^3^University Institute for Diagnostic, Interventional and Pediatric Radiology, Inselspital, University Hospital Bern, University of Bern, Bern, Switzerland; ^4^Department of Radiation Oncology, Inselspital, Bern University Hospital, University of Bern, Bern, Switzerland; ^5^Mimacom AG, Bern, Switzerland

**Keywords:** medical history taking, conversational agent, consumer health information, algorithms, patients, radiology, user-centered design, natural language processing

In the published article, there was an error in the Data availability statement. We were missing to add the links to the repositories mentioned in the paper. The correct Data availability statement appears below.

The original contributions presented in the study are available https://doi.org/10.17605/OSF.IO/6MBHD (MIA questionnaire and FHIR resources), https://doi.org/10.5281/zenodo.10782322 (code for the chatbot) and are included in the article/Supplementary material, further inquiries can be directed to the corresponding author.

The authors apologize for this error and state that this does not change the scientific conclusions of the article in any way. The original article has been updated.

