# Supplementary material for: Person-based design and evaluation of MIA, a digital medical interview assistant for radiology
Source: Front Artif Intell. 2024 Aug 16;7:1431156. doi: 10.3389/frai.2024.1431156 (PMC11363708; doi:10.3389/frai.2024.1431156)
Supplement: Supplementary file 1 [file Data_Sheet_1.pdf]

Supplementary Material

1 USABILITY QUESTIONS

The table lists the BUS-11 questions for assessing the usability.

Factors	Items
**Perceived accessibility to chatbot functions**	Q1: The chatbot function was easily detectable.<br>Q2: It was easy to find the chatbot.
**Perceived quality of chatbot functions**	Q3: Communicating with the chatbot was clear.<br>Q4: The chatbot was able to keep track of context.<br>Q5: The chatbot's responses were easy to understand.
**Perceived quality of conversation and information provided**	Q6:I find that the chatbot understands what I want and helps me achieve my goal.<br>Q7: The chatbot gives me the appropriate amount of information.<br>Q8: The chatbot only gives me the information I need.<br>Q9: I feel like the chatbot's responses were accurate.
**Perceived privacy and security**	Q10: I believe the chatbot informs me of any possibly privacy issues.
**Time response**	Q11: My waiting time for a response from the chatbot was short.

Table S1. BUS-11: Bot usability scale

The table lists questions that we added to assess the usability. They result from the evaluation framework applied as basis for the evaluation.

Q1: I have the impression that the digital assistant understands what I want to know.
Q2: I have the impression that the digital assistant helps me to get answers to my questions about the examination.
Q3: The digital assistant provides me with an appropriate amount of information.
Q4: I have the feeling that the digital assistant's answers are tailored to my needs.
Q5: The waiting time for a response from the digital assistant was in line with my expectations.
Q6: The digital assistant did not recognize how much I was bothered by some of the things discussed.
Q7: The digital assistant understood my words but not my feelings.
Q8: I think the digital assistant generally understood everything I said.
Q9: I like the background color.
Q10: The font was easy to read.
Q11: The font size was appropriate for me.

Table S2. Additional usability questions

2 HEURISTICS

In the table, the heuristics underlying the heuristic evaluation are described.

**Heuristic criteria**	**Expectations exceeded (3 points)**	**Expectations reached (2 points)**	**Below expectations (1 point)**
Visibility of system status	The system always informs the user about what is happening - in good time and with appropriate feedback.	The system informs the user about what is currently happening - sometimes with a delay or without feedback.	The system rarely or never provides information about what is happening. Feedback is non-existent or misleading.
Match between system and real world	The system speaks and understands the language of the user - with words, sentences and concepts that are familiar to the user (e.g. no technical terms). The information appears in a natural and logical order. The dialogue is conducted in such a way that the conversation starts and ends smoothly and is conducted appropriately.	The system mainly speaks and understands the language of the user - with words, sentences and concepts that are familiar to the user (e.g. no technical terms). The information appears in a natural and logical order. The dialogue is conducted in such a way that the conversation starts and ends smoothly and is conducted appropriately.	The system does not speak or understand the user's language - words, sentences and concepts are usually unfamiliar to the user. The information is not presented logically. The dialogue seems unnatural.
User control and freedom	The user often carries out actions unintentionally. The system offers ways to easily exit the state reached, especially without having to go through a long dialogue. Exits such as "Undo", "Redo" and "Cancel" are therefore always possible and visible.	The user often carries out actions unintentionally. The system offers partially ways to easily exit the state reached, especially without having to go through a long dialogue. Exits such as "Undo", "Redo" and "Cancel" are therefore often possible and visible.	The user often carries out actions unintentionally. The system offers no ways to easily exit the state reached, or only through a long dialogue. Exits such as "Undo", "Redo" and "Cancel" are impossible or barely visible.
Consistency and standards	The user does not have to think about whether different words, situations and actions mean the same thing. The user always receives consistent answers when they ask the same question in different ways. The system has a consistent language style and personality within the interaction.	The user does not have to think about whether different words, situations and actions mean the same thing. The user often receives consistent answers when they ask the same question in different ways. The system has often a consistent language style and personality within the interaction.	The user must consider whether different words, situations and actions mean the same thing. The user does not receive standardised answers if they ask the same question in different ways. The system does not have a consistent language style and personality within the interaction.
Error prevention	The dialogue is designed in such a way that no problems in the interaction occur. The dialogue runs undisturbed, without interruptions and pauses. Error messages do not occur. The system avoids error-prone situations situations or warns the user user before or in the event of misuse.	The dialogue is designed in such a way that often no problems in the interaction occur. The dialogue mostly runs undisturbed, only with few interruptions and pauses. The system avoids error-prone situations situations or warns the user user before or in the event of misuse.	The dialogue is disrupted and only possible with interruptions and pauses. Error messages occur frequently. The system favours error-prone situations. It does not warn the user before or in the event of incorrect use.

Help and guidance	The system accompanies the user user during the entire dialogue dialogue by clarifying the possibilities of the system. The help functions are easy to find, focus on the user's task the user's task, list specific steps and concrete steps and are not too extensive. Possible actions and options are explained.	The system partially accompanies the user during the dialogue by clarifying the possibilities of the system. The help functions are easy to find, focus on the user's task, usually list specific steps and are not too extensive. Possible actions and options are usually explained.	The system does not accompany the user during the dialogue. The system's options are not made clear. The help functions cannot be found, do not list specific steps or are too extensive. Possible actions and options are not explained.
Flexibility and efficiency of use	The system supports flexible interactions. Shortcuts and other shortcuts - invisible to new users - speed up operation for advanced users. Frequent actions can also be customised.	The system mostly supports flexible interactions. Shortcuts and other shortcuts - invisible to new users - speed up operation for advanced users. Frequent actions can also be customised.	There are no shortcuts or other shortcuts to make operation easier.
Aesthetic, minimalist, and engaging design	Dialogues do not contain any information that is irrelevant or rarely needed. The system supports short interactions. A more extensive conversation is possible if the user so wishes.	Dialogues do not contain any information that is irrelevant or rarely needed. The system supports short interactions. A more extensive conversation is sometimes possible if the user so wishes.	Dialogues contain information that is irrelevant or rarely needed. The system does not support short interactions. A more comprehensive conversation is not possible.
Help users recognize, diagnose, and recover from errors	Error messages are formulated in simple language. They state the problem precisely and suggest a constructive solution.	Error messages are sometimes formulated in simple language. They usually state the exact problem or suggest a constructive solution.	Error messages are not clearly formulated. They do not name the problem and do not suggest a constructive solution.
Context preservation	The context in relation to the topic of conversation is retained within the dialogue. The user has the option of referring to previous messages in further interactions.	The context in relation to the topic of conversation is retained within the dialogue. The user does not have the option of referring to previous messages in further interactions.	The context in relation to the topic of conversation is not retained within the dialogue. The user has no option to refer to previous messages in further interactions.
Trustworthiness	The system conveys trustworthiness by ensuring the confidentiality of user data and being transparent and truthful to the user in this regard. The system does not falsely claim to be a human being.	The system explains to the user what happens to the data. The system does not falsely claim to be a human being.	The system does not convey trustworthiness. The use of user data is not transparent. The system claims to be human.

Table S3. Heuristics adapted from Langevin et al.

3 EVALUATION FRAMEWORK

In the following, the results for the complete evaluation framework are summarized. Judgments from study participants were aggregated in a way that number of votings ”strongly agree” and ”agree” were merged and number of votings ”strongly disagree” and ”disagree” were merged. All values related to the exploratory user study refer to n=36 participants.

Evaluation category	Metric	Method	Result
**Accessibility**	What is the readability level of the health CA content?	Readability checker: https://www.supertext.ch/tools/lix, https://www.flesch-lesbarkeitsindex.de, https://charactercalculator.com/gunning-fog-index, https://charactercalculator.com/smog-readability	LIX: Interview module has language level C2, Q&A module has language level C1; Flesch Reading Score, Gunning Fog Index and SMOG: Interview module content is in plain English readability and easy understandable or fairly difficult to read (Gunning Fog Index). Content provided by the Q&A module is difficult to read, corresponding to a college or undergraduate reading level.
	What is the health literacy of the user?	Dropped	n/a
	What is the required health literacy level for using the health CA?	Dropped	n/a
	Does the health CA provide alternatives for written in- and output (e.g. icons, images, voice as text alternatives)?	Design and implementation check	No
	Is the contrast between text and background color at least 4.5:1?	Design and implementation check	No (3.6:1)
	Is it possible to resize the text?	Design and implementation check	Yes
	Are accessibility guidelines of the used service channel applied (e.g. Web Content Accessibility Guidelines, Android / Apple Accessibility Guidelines)?	Design and implementation check	No
**Ease of use**	Technical issues	Comments from think aloud	see description of usability test
	Usability assessment using the BUS as a standard means within CA evaluation	Exploratory user study (all participants)	see description of usability test
	Considering the suggested 11 heuristic criteria for health CA design	Exploratory user study (only participants from patient lobby group	21.9/33 with n=6 participants, see description of usability test
**Engagement**	Goal / task completion rate	Dropped	n/a
	Retention rate	Dropped	n/a
	Speed	Dropped	n/a
	Satisfaction	Dropped	n/a
	Dialogue efficiency	Dropped	n/a
**Classifier performance**	Precision	Calculated for Q&A module	0.51
	Recall	Calculated for Q&A module	0.87
	F-Score	Calculated for Q&A module	0.64
	Accuracy	Calculated for Q&A module	0.54
**Flexibility in dialogue handling**	Can the health CA deal with answers to questions that give more information than was requested?	Design and implementation check	no
	Can the health CA deal with answers to questions that give different information than was requested?	Design and implementation check	no

**Flexibility in dialogue handling**	Can the health CA reformulate an utterance on request?	Design and implementation check	no
	Can the health CA deal with answers to questions that give less information than was actually request	Design and implementation check	no
	Can the health CA deal with negatively specified information?	Design and implementation check	no
	Can the health CA deal with 'help' sub-dialogues initiated by the user?	Design and implementation check	no
	Does the health CA deal with 'non-help' sub-dialogues initiated by the user?	Design and implementation check	yes
	Can the health CA deal with inconsistent information?	Design and implementation check	no
**Content accuracy**	Is the underlying knowledge base evidence-based (e.g. appropriate peer reviewed scientific literature used)?	Design and implementation check	yes
	Were healthcare professionals involved in the content development of the health CA?	Design and implementation check	yes
	Is there a maintenance process for the information included in the health CA?	Design and implementation check	no
	Is information on the developer or content provider of the health CA provided?	Design and implementation check	yes, during the on-boarding process
	Were patient organizations involved in the development of the health CA?	Design and implementation check	yes
**Context awareness**	Does the health CA reliably recognize context switches?	Dropped	n/a
	Is the health CA able to clarify the context when it is not clearly formulated?	Dropped	n/a
	Is the CA using personal user data to contextualize the request/question and generate the answers?	Design and implementation check	no
**Error tolerance**	Fallback rate	Dropped	n/a
**Security**	Is ISO/IEC 27001 or another recognized standard related to information security management applied?	Design and implementation check	no
	Is an assessment of information security risks and potential consequences available?	Design and implementation check	The packages used in the app are scanned for vulnerabilities automatically.
	Was a secure-by-design process pursued?	Design and implementation check	no
	Are processes or measures in place for managing reliability and maintenance of third party software and components used in the health CA?	Design and implementation check	no

**Security**	Is the security of the health CA tested on a regular basis?	Design and implementation check	no
	Is a process to prevent unauthorized access and modification to the source code and knowledge base of the health CA in place?	Design and implementation check	no
	Is an information security policy available for the user?	Design and implementation check	no
	Is a process in place of reporting, identifying, assessing, logging and responding to security vulnerabilities?	Design and implementation check	yes
	Is data encryption used for encrypting user data?	Design and implementation check	no
	Is user authentication, authorization and session management implemented?	Design and implementation check	yes
	Are standard operating procedures in place for processing personal identifiable information according to the privacy statement?	Design and implementation check	yes
	Is a privacy statement available for the user?	Design and implementation check	yes, in the on-boarding
	Is the health CA compliant with the current regulations about data privacy (e.g. GDPR in Europe and UK and HIPAA in US)?	Design and implementation check	yes
**Appropriateness of responses**	Proportion of appropriate responses to users' questions or answers	Dropped	n/a
	I find that the CA understands what I want and helps me achieve my goal.	Exploratory user study	17% disagree, 67% agree
	The CA gives me the appropriate amount of information.	Exploratory user study	11% disagree, 67% agree
	The CA only gives me the information I need.	Exploratory user study	14% disagree, 61% agree
	I feel like the CA's responses were accurate.	Exploratory user study	25%disagree, 56% agree
	I feel like the CA's responses (information) were adapted to my characteristics/conditions.	Exploratory user study	17% disagree, 50% agree
	The CA gives me some relevant suggestions that provide me with additional relevant information. (adapted to: I believe that the CA supports me in getting answers to my questions related to the examination.)	Exploratory user study	14% disagree, 61% agree
**Comprehensibility**	Communicating with the CA was clear.	Exploratory user study	0% disagree, 86% agree
	The CA was able to keep track of context.	Exploratory user study	17% disagree, 72% agree
	The CA's responses were easy to understand.	Exploratory user study	8% disagree, 83% agree

**Speed of response**	Average time needed for a CA to post a reply.<br>My waiting time for a response from the CA was aligned with my expectation.	Dropped<br><br>Exploratory user study	n/a<br><br>3% disagree, 92% agree
**Empathy**	Does the CA include techniques for sentiment and emotion analysis?<br>The CA appreciated what my experiences feel like to me.<br>The CA did not realize how strongly I fell about some of the things we discussed.<br>The CA understood my words, but not the way I feel.	Design and implementation test<br><br>Dropped<br><br>Exploratory user study<br><br><br>Exploratory user study	no<br><br><br>n/a<br><br>39% disagree, 33% agree<br><br><br>22% disagree, 42% agree
**Linguistic accuracy**	Percentage of grammatically incorrect sentences<br>Percentage of grammatically incorrect words<br>Percentage of wrong use of terms<br>I feel like the CA‘s responses were accurate.	Dropped<br><br>Dropped<br><br>Dropped<br><br>Exploratory user study	n/a<br><br>n/a<br><br>n/a<br><br>25% disagree, 56% agree
**Under-standing**	I think the CA understood me.<br>I think the CA usually understood all of what I said to him or her.	Exploratory user study<br><br>Exploratory user study	25% disagree, 58% agree<br><br>14% disagree, 64% agree
**Background color and content**	I like the background color.	Exploratory user study	8% disagree, 64% agree
**Font type and size**	The font type of the CA was well readable.<br>The font size was appropriate to me.	Exploratory user study<br><br>Exploratory user study	6% disagree, 89% agree<br><br>6% disagree, 89% agree
**Button color, shape, icon**	I liked the button color.<br>I liked the button shape.<br>Understanding the icons was easy.	Dropped<br>Dropped<br>Dropped	n/a<br>n/a<br>n/a

Table S4. Detailed results from applying the evaluation framework
